# Linking Cortical Morphology and Neurophysiological Dynamics in Parkinson’s Disease

**DOI:** 10.21203/rs.3.rs-7992155/v1

**Published:** 2025-11-24

**Authors:** Koorosh Mirpour, Amirreza Alijanpourotaghsara, Nader Pouratian

**Affiliations:** 1 Department of Neurological Surgery, University of Texas Southwestern Medical Center, Dallas

**Keywords:** Parkinson’s disease, Deep Brain Stimulation, DBS, Beta Oscillation, Beta Burst, GPi, Motor Cortex, Cortical morphometry, cortical structure, electrophysiological biomarkers, neuroimaging, structural MRI, Gray matter thickness, Gray matter volume, Local field potential, LFP, ECoG

## Abstract

Parkinson’s disease (PD) involves progressive neurodegeneration and distinctive structural and functional alterations in cortico-basal ganglia circuits. This study proposes bridging the gap between structural and functional biomarkers to uncover fundamental mechanisms underlying PD pathophysiology and support more comprehensive diagnostic and therapeutic approaches.

This study analyzed intraoperative electrocorticography (ECoG) and pallidal (GPi) local field potential (LFP) recordings alongside preoperative T1-weighted structural MRI from 50 patients with PD undergoing deep brain stimulation surgery. We extracted 36 morphometric features (thickness, volume, surface area) from nine sensorimotor Brodmann areas using FreeSurfer and 92 neurophysiological features (power, burst dynamics, coherence) across multiple frequency bands.

Pairwise analyses revealed only fragmented, though significant, correlations. In contrast, the SPLS analysis identified a robust and significant latent dimension (test set rho = 0.818, p = 0.001) linking the two modalities. This primary latent variable was driven by a strong negative association with cortical thickness in sensorimotor areas (e.g., BA3b, BA1, BA6) and a complex combination of neurophysiological features, most notably altered burst dynamics in the alpha, gamma, and low-beta bands. This structure-function relationship was independent of age, disease duration, and UPDRS-III scores (rho = 0.701 after partialling out confounds). Critically, the SPLS model failed to find a significant correlation when applied to the ET cohort (rho = −0.342, p = 0.102), suggesting the identified relationship is specific to PD pathophysiology.

These findings highlight the value of multimodal approaches for uncovering structure–function interactions in PD, and the potential of integrated biomarkers for improving diagnosis and treatment strategies.

## Introduction

Parkinson’s disease (PD) Pathophysiology involves multifaceted changes in brain structure and function, which have largely been investigated independently via neuroimaging (morphometry) and electrophysiology (neural oscillations). Electrophysiological investigations have highlighted abnormal neural oscillations within the basal ganglia circuitry of people with PD (PwPD), particularly exaggerated beta-frequency oscillatory activity (13–35 Hz) in the subthalamic nucleus (STN) and globus pallidus internus (GPi) (Brown et al., 2001; Malekmohammadi et al., 2018a). This heightened beta power correlates with motor symptoms such as rigidity and bradykinesia and decreases during movement preparation and execution (Kühn et al., 2004; Litvak et al., 2012)and is modulated by treatments such as deep brain stimulation (DBS), with beta suppression tracking motor improvement (Neumann et al., 2016; Oswal et al., 2016). These suggest a direct link between aberrant electrophysiological patterns and the hypodopaminergic state.

The beta band is often subdivided into low beta (13–20 Hz) and high beta (21–35 Hz) frequencies (Little et al., 2012; Hirschmann et al., 2019). The low beta band particularly in STN appears more pathological and medication sensitive, while high beta band is less responsive to medication (Neumann et al., 2016) and may have a broader role across the BGTC motor circuit (Malekmohammadi et al., 2018b). Beyond average power, spectral coherence within the cortico-basal ganglia network (Oswal et al., 2016; Van Wijk et al., 2017; Malekmohammadi et al., 2018b) and transient beta bursts (Tinkhauser et al., 2017; Lofredi et al., 2019; O’Keeffe et al., 2020) have emerged as crucial biomarkers correlating with motor impairment

In parallel, neuroimaging studies documented progressive brain structural changes in PwPD. such as cortical thinning and gray matter (GM) volume loss, which often correlates with disease severity (e.g., UPDRS-III scores) (Pereira et al., 2014; Zhang et al., 2018; Tessa et al., 2014; Jia et al., 2015). However, inconsistencies complicate the interpretation (Brenneis et al., 2003; Weintraub, 2011; Burton, 2004; Morgen et al., 2011; and despite these advances, the relationship between neurophysiology and structural brain changes in PwPD remains inadequately explored.

A few studies have begun to bridge this gap. (Sanmartino et al., 2022) proposed links between STN beta power and neurodegeneration in the striatum and prefrontal regions. Our group recently found a significant negative correlation between high-beta (20–35 Hz) power and cortical thickness, suggesting that cortical thinning may contribute to elevated high-beta activity (Cohen et al., 2023).

Understanding the interplay between neurophysiology (an emergent property of underlying neural activity) and cortical morphometrics (a context for physiological change) is crucial for new insight into the pathophysiology of the cortico-basal ganglia circuitry in PD. One major obstacle is the disease’s multifactorial nature, involving complex interactions between multiple electrophysiological and structural markers. Traditional pairwise analyses may be insufficient to capture these comprehensive associations.

We hypothesize that a general link exists between electrophysiology and cortical morphometrics that extends beyond isolated pairwise correlations between individual biomarkers. We propose that dynamic and complex electrophysiological processes (e.g., bursting, coherence) are correlated collectively with structural alterations. We believe that distributed biomarkers from both modalities are linked to fundamental core pathophysiological mechanisms that are equally associated with the distributed neurophysiology of the disease and that sparse, pairwise correlations fail to capture the interconnected and holistic nature of these core mechanisms.

Our aim is to explore this intricate relationship. We will investigate the associations between local cortical thickness, surface area, and volume, and their cross-correlations with neurophysiological biomarkers.

To move beyond sparse correlations and capture holistic interactions, we will employ advanced multivariate methods, specifically canonical correlation analysis (CCA). This approach will reduce the high-dimensional biomarkers from into low-dimensional latent variables, allowing us to maximize the covariation between structure and neurophysiology. This focus on associative effects, distinct from correlations with clinical features (e.g., age, disease duration), strives to unravel the fundamental interplay between electrophysiological activity and cortical morphometrics. Elucidating these complex interactions may identify novel biomarkers and enhance our understanding of PD pathophysiology.

## Methods

### Participants and Ethical Approval

This study was approved by the Institutional Review Boards of both the University of California, Los Angeles (UCLA), and UT Southwestern Medical Center at Dallas. To increase the overall statistical power, data from both centers were pooled for the final analysis, as initial evaluations confirmed that the principal findings were consistent across sites. All participants provided written informed consent in accordance with the Declaration of Helsinki. Deep Brain Stimulation (DBS) implantations and target selection were based solely on clinical grounds and indication, based on recommendations of an interdisciplinary clinical team.

Data was derived from 50 patients diagnosed with PD who underwent DBS implantation surgery. Bilateral GPi leads were implanted in all but five patients, who received unilateral implantations (3 left, and 2 right). Detailed patient demographics are provided in Table 1. To adhere to standard clinical protocols, all patients presented for surgery in a practical off-state, with PD medications withheld for at least 12 hours prior to the start of surgery.

### Surgical Procedure and Electrophysiological Recordings

**Figure 1:**
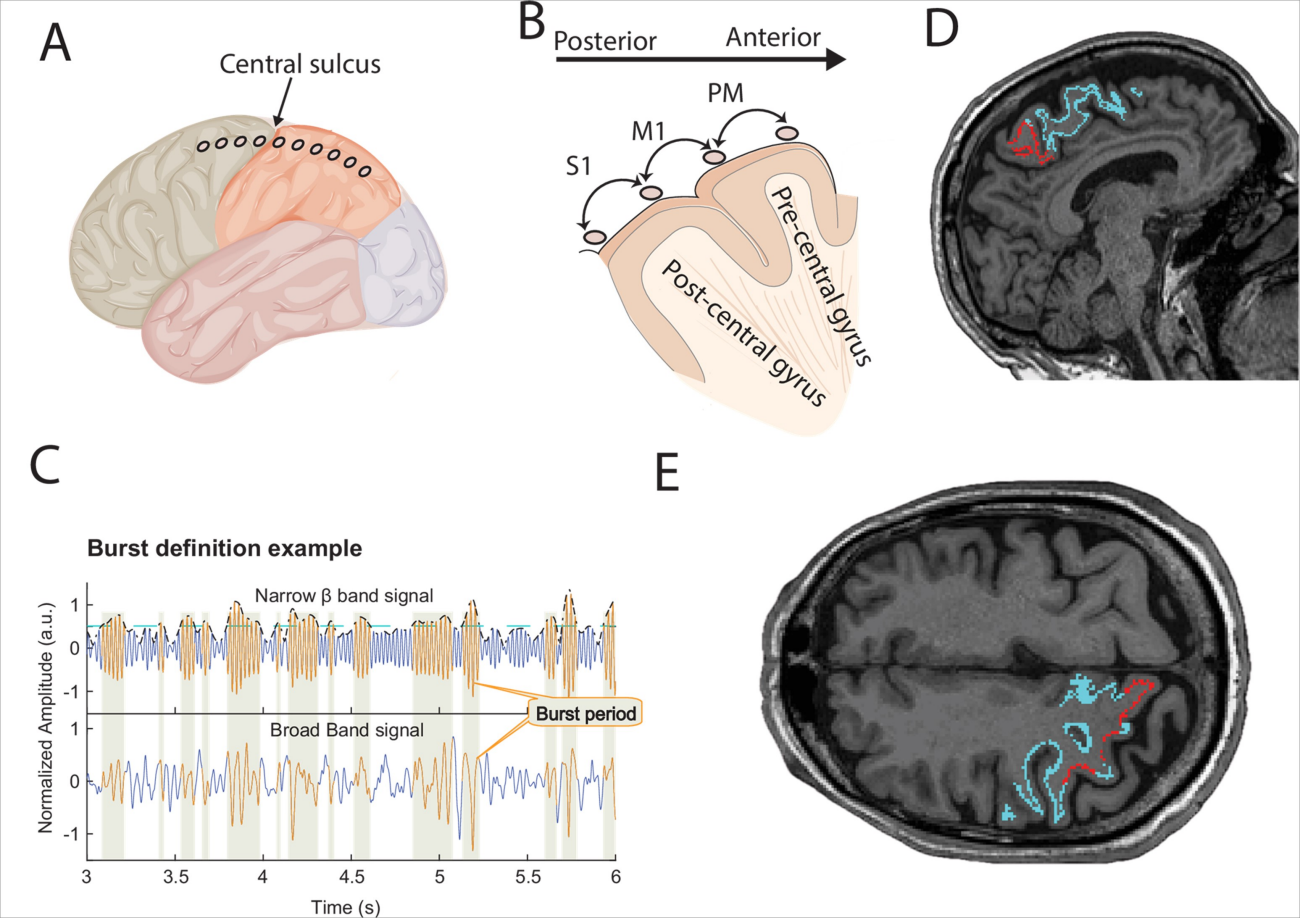
Methodology of the study (A and B) Schematic illustration of the location of an ECoG strip on the cortical surface, with electrode contacts marked as red dots in (A). The central sulcus (M1, black line in A) serves as the structural boundary. The bipolar contact spanning both sides of the central sulcus corresponds to the M1. The first bipolar contact pair anterior to the M1 is designated as PM (Premotor cortex), while the first bipolar contact pair posterior to the M1 is labeled as S1 (Primary somatosensory cortex). (C) An example of burst analysis. Burst periods are highlighted on the filtered narrowband beta band signal (blue trace), the analytical envelope of the signal, and the 75^th^ percentile threshold (green dashed line). The burst periods are marked (orange trace) on the bottom panel’s original broadband signal (blue trace). (D & E) High-resolution T1-weighted MRI figures from subject bG10 showcase the anatomical primary motor region, Brodmann Area 4a (BA4a, red line), and the PM region (Brodmann Area 6, BA6, blue line) using FreeSurfer’s Recon-all command for parcellation.

Following preoperative clinical stereotactic planning, burr holes were created for DBS lead placement. A subdural electrocorticography (ECoG) strip with eight contacts (4 mm platinumiridium electrodes spaced 1 cm apart; AdTech Medical) was inserted subdurally in a posterior direction through the burr hole. Once a clinical baseline was established after discontinuation of propofol, DBS leads were implanted using microelectrode recordings, kinesthetic mapping, and macroelectrode stimulation testing. Experimental recordings from the deep brain leads and ECoGs (Figure 1A) were performed simultaneously while patients were awake, at least 30 minutes after cessation of propofol sedation.

Neurophysiological recordings were conducted at a sampling rate of 2,400 Hz at UCLA and 1,375 Hz at UT Southwestern (UTSW), with a bandpass filter set between 0.1 and 1,000 Hz. At UCLA, recordings utilized the BCI2000 v3 system connected to amplifiers (g.Tec, g.USBamp 2.0), and at UTSW, the NeuroOmega system was used, with a single scalp reference electrode. Electrode locations were determined postoperatively through a combination of preoperative magnetic resonance imaging (MRI), intraoperative fluoroscopy, and postoperative CT scans, following established protocols (Randazzo et al., 2016; Malekmohammadi et al., 2018a, 2018b). The electrode contact located anterior to the central sulcus was designated as overlying the precentral sulcus (primary motor cortex), while the contact immediately anterior to this was identified as corresponding to the premotor cortex (Figure 1B).

### Imaging Acquisition and Processing

High-resolution pre-contrast T1-weighted anatomical scans were acquired on 3T scanners at two imaging centers. At UCLA, imaging was performed using a Siemens Skyra (syngo MR D13D) with sagittal 3D acquisition parameters: TR = 2100 ms, TE = 2.44 ms, TI = 1100 ms, flip angle = 15°, slice thickness = 1.0 mm, matrix = 256 × 256, in-plane acceleration factor = 2, and pixel bandwidth = 240 Hz/pixel. At UTSW, a Philips Ingenia (software version 5.7.1) was used with axial 3D acquisition: TR = 7.67 ms, TE = 3.41 ms, flip angle = 8°, slice thickness = 0.7 mm with 0.35 mm spacing, acquisition matrix = 301 (reconstructed to 512), parallel imaging acceleration = 2 × 2, pixel bandwidth = 241 Hz/pixel, estimated echo spacing = 8.1 μs, and total readout time = 4.14 ms.

In addition to T1-weighted imaging, all participants underwent standard T2-weighted (T2W) imaging and either a Fast Gray Matter Acquisition T1 Inversion Recovery (FGATIR) sequence or a subthalamic nucleus (STN)-optimized T2-weighted acquisition, depending on the center-specific protocol. T1-weighted anatomical scans were processed using FreeSurfer (version 7.3.2; Fischl, 2012) for cortical reconstruction and parcellation. The standard pipeline included skull stripping, intensity normalization, Talairach registration, subcortical segmentation, cortical surface reconstruction, and anatomical labeling in each subject’s native space.

Cortical parcellation was performed using FreeSurfer’s ex vivo Brodmann Area Maps, a probabilistic cytoarchitectonic atlas derived from histologically defined regions in ten postmortem human brains (Fischl et al., 2008). We focused on nine Brodmann areas relevant to sensorimotor and language functions: BA1, BA2, BA3a, BA3b (somatosensory), BA4a, BA4p (primary motor), BA6 (premotor), and BA44, BA45 (Broca’s area). These regions were selected a priori as they form the core of the sensorimotor network and have well-established roles in motor, somatosensory, and speech-language functions, all of which are frequently impacted in Parkinson’s disease (Chu et al., 2024). By targeting the regions defined in the cytoarchitectonic atlas (Fischl et al., 2008), we could directly investigate the relationship between the structure of these clinically relevant areas and their underlying neurophysiology. For each region, we extracted four morphometric features: mean cortical thickness, surface area, cortical volume, and standard deviation of thickness. This resulted in 36 imaging-derived features per subject.

Quality control of cortical parcellations was performed through detailed visual inspection of the reconstructed cortical surfaces and Brodmann area label overlays. For each subject, both inflated and pial surfaces were reviewed using FreeSurfer’s visualization tools to assess the anatomical accuracy of surface reconstruction and regional label placement, with particular focus on sensorimotor regions around the central sulcus. All subjects passed visual QC criteria, and no manual edits or exclusions were required.

No correction for intracranial volume (ICV) was applied to the morphometric data. This decision was based on our primary analytical goal, which was to examine the direct, within-cohort associations between local cortical structure and local neurophysiology, rather than to perform between-group volumetric comparisons where ICV is a critical covariate. Furthermore, applying a uniform global correction would be methodologically inappropriate for our specific structural metrics. Cortical thickness, a key variable in our analysis, is largely independent of ICV and is not typically corrected in standard pipelines (Schwarz et al., 2016; Dhamala et al., 2022).

### Data Analysis

All analyses were performed offline using custom-made scripts in MATLAB (MathWorks Inc., Natick, MA, USA).

#### Neurophysiological Data Preprocessing

Local field potentials within the GPi were recorded in a bipolar configuration using adjacent contacts. All data analyzed were collected during DBS-OFF states where subjects maintained a minimum of at least 15 seconds of continuous resting without unnecessary voluntary movements.

Three bipolar ECoG signal pairs were defined:

##### Premotor cortex (PM):

Contacts immediately anterior to the central sulcus.

##### Motor cortex (M1):

Contacts located on either side of the central sulcus.

##### Primary somatosensory cortex (S1):

Contacts immediately posterior to the central sulcus (Figure 1B).

Neurophysiological data preprocessing included removal of 60 Hz line noise and discarding segments with electrical or movement artifacts, following previously published procedures (AuYong et al., 2018). Artifact segments were identified based on criteria such as abnormally high power spectral values, excessive harmonics, and rapid voltage changes. Artifact removal was automated through full-wave rectification, calculation of the first derivative, and application of a five-point median filter. Segments where the first derivative exceeded five Median Absolute Deviation (MAD) of the subject’s entire recording were considered artifacts and replaced by linear interpolation using data from 2 ms before and after the artifact. Less than 0.1% of the data was corrected using this method.

Power spectral density (PSD) was estimated using Thomson’s multitaper method (Thomson, 1982) with 1-second consecutive, non-overlapping time windows across a frequency range of 4 to 110 Hz. A frequency bandwidth of ±2 Hz and three tapers were used. To control for inter-subject variability in baseline power, each segmented spectrum was normalized to the total power of the signal for each condition, excluding line noise and its harmonics. Average power within specific frequency bands—alpha (8–11 Hz), low beta (12–20 Hz), high beta (21–35 Hz), and high gamma (65–110 Hz)—was calculated using the multitaper PSDs.

**Figure 2:**
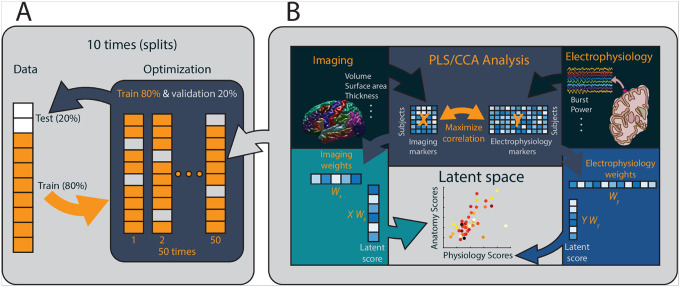
Schematic pipeline of associative correlation analysis (A) The analysis method illustrated in the figure follows a comprehensive multiple-holdout framework. This approach begins by splitting the original dataset into two parts: an optimization set (comprising 80% of the data) and a holdout set (comprising the remaining 20%) as shown in panel A. The optimization set is divided into a training set (80%) and a validation set (20%). These subsets are used to fit a regularized Partial Least Squares/Canonical Correlation Analysis (PLS/CCA) model and to optimize the regularization parameters through 50 different training and validation iterations. After determining the optimal parameters, the regularized PLS/CCA model is refitted using the entire optimization set and then evaluated on the holdout set with permutation testing to assess its performance. This whole process is repeated 10 times to enhance the robustness and reliability of the model results. (B) The panel B illustrates the PLS/CCA models, which are employed to find weight vectors that maximize the covariance (in the case of PLS) or correlation (in the case of CCA) between linear combinations of brain imaging and behavioral data. The sparse PLS model introduces sparsity constraints, effectively reducing some weights for imaging and electrophysiology variables to zero, thereby selecting the most significant features. The resulting linear combinations or weighted sums of structural and neurophysiological data (derived from matrices *X* and *Y* with corresponding weights Wx and Wy) yield structure and neurophysiology scores (*X*Wx and *Y*Wy) for each participant. These scores are then used to construct an structure-neurophysiology latent space, which represents the relationships between structural and neurophysiological factors across the entire study sample.

Large matrices containing structural and neurophysiological data were subsequently constructed and prepared for cross-correlation analysis and Sparse Partial Least Squares (SPLS) analysis (Figure 2).

#### Feature Extraction

Bursting periods were defined as oscillation amplitude periods exceeding the 75th percentile of power for that subject within a specific frequency band and persisting for a minimum of one complete cycle (Figure 1C). We computed the average burst duration, burst amplitude, and burst rate (number of bursts per second) for each subject. To quantify the degree of co-variability between cortico-cortical and cortico-subcortical signal pairs, magnitude-squared coherence was estimated using the multitaper method with the same time window and frequency smoothing parameters as the PSD analysis (±2 Hz frequency bandwidth with three tapers).

For each patient, a total of 92 electrophysiological measures were calculated across five frequency bands and four recording sites (Motor cortex (M1), Premotor cortex (PM), Primary somatosensory cortex (S1), and pallidal electrodes). These measures included power, burst duration, burst amplitude, burst rate, cortico-cortical coherence, and cortico-subcortical coherence. Cortico-cortical coherences were calculated for three pairs between the central ECoG and the other cortical ECoGs (PM and S1), and cortico-subcortical coherences were calculated between the pallidal lead with the highest average beta power and the three cortical ECoGs.

### Statistical Analysis

#### Pairwise Cross-Correlation Analysis

We examined the relationships between imaging and neurophysiological measures by calculating Pearson’s correlation coefficients for all possible pairs and organizing the results based on subfeatures of each set. Structural measures were divided into two subgroups:

##### Brain regions:

Brodmann areas around the general regions where ECoG electrodes were placed, specifically BA1, BA2, BA3a, BA3b, BA4a, BA4p, BA6, BA44, and BA45.

##### Morphometric parameters:

Average surface area, volume, average thickness, Neurophysiological measures were categorized into three subgroups:

##### Electrode locations:

M1, Premotor cortex, Primary somatosensory cortex, and subcortical sites.

##### Neurophysiological metrics:

Power, burst duration, burst amplitude, burst rate, cortico-cortical coherence (CC), and cortico-subcortical coherence (M1).

##### Frequency bands:

Alpha, low beta, high beta, and gamma.

**Figure 3:**
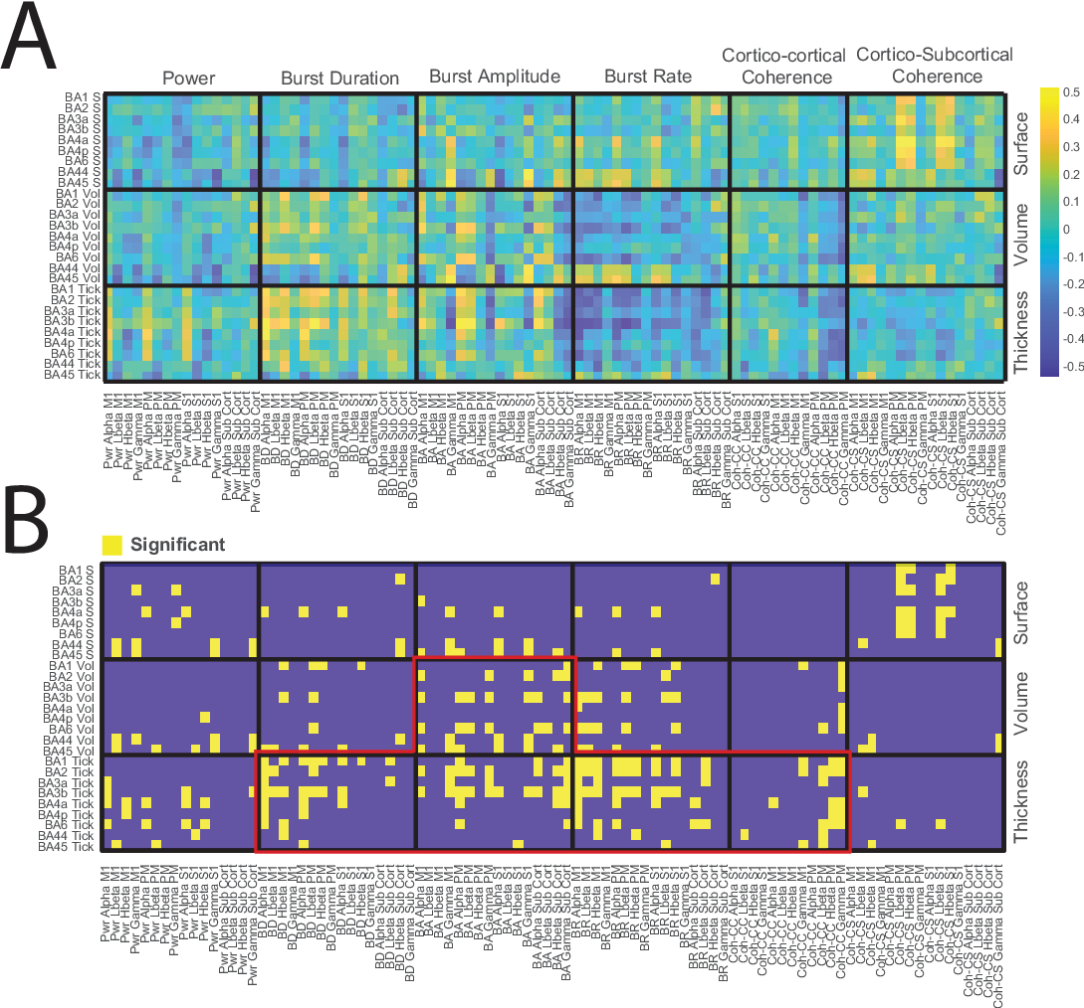
Cross-Correlation Analysis Between Structural and Electrophysiological Measures in PD Patients. (A) Heatmap showing the pairwise Pearson correlation coefficients between structural and electrophysiological measures across 50 PD patients. The structural measures include cortical thickness, volume, and surface area for Brodmann areas (BA1, BA2, BA3a, BA3b, BA4a, BA4p, BA6, BA44, BA45), while the electrophysiological measures encompass power, burst duration, burst amplitude, burst rate, cortico-cortical coherence, and cortico-subcortical coherence, calculated across various frequency bands. The color scale represents the strength and direction of the correlation coefficients. (B) The significance map of the correlations is displayed in (A) following permutation testing. Significant correlations (p < 0.05) are highlighted in yellow. Red outlines indicate clusters where the significance exceeds three median absolute deviations from the center of the distribution. The figure emphasizes regions of significant association between structural brain metrics and electrophysiological activity, particularly in the areas related to burst dynamics and cortical thickness. BA = Broadman area, S = surface area, Vol = volume, Tick = average thickness, Pwr = power, LBeta = low beta band, Hbeta = high beta band, BD = burst duration, BA = Burst amplitude, BR = burst rate, M1 = Motor cortex, PM = Premotor cortex, S1 = Primary somatosensory cortex, Sub cort = subcortical region (Pallidum), Coh-CC = cortico-cortical coherence, Coh-M1 = cortico-subcortical coherence.

**Figure 4:**
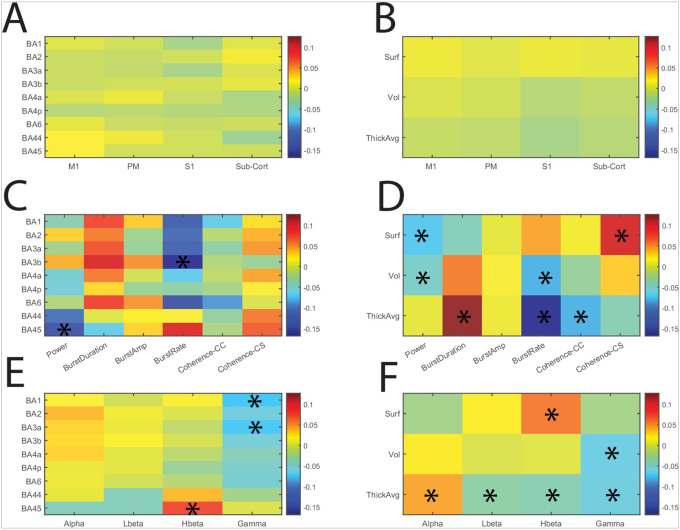
Detailed Analysis of Cross-Correlation Between Structural and Electrophysiological Measures. (A-F) Heatmaps illustrate the pairwise Pearson correlation coefficients between structural and electrophysiological measures, grouped by different subcategories across 50 PD patients. The color scale represents the strength and direction of correlation coefficients. Asterisks (*) indicate significant correlations after permutation testing and False Discovery Rate (FDR) control. (A) Correlation of structural measures (BA1 to BA6) with electrophysiological metrics categorized by electrode location: M1, PM and S1, and subcortical (Sub-Cort). (B) Correlation of surface area (Surf), volume (Vol), and average thickness (Thick Avg) with electrophysiological metrics categorized by electrode location. (C) Correlation of structural measures (BA1 to BA6) with detailed electrophysiological metrics: power, burst duration, burst amplitude, burst rate, cortico-cortical coherence (Coherence-CC), and cortico-subcortical coherence (Coherence-M1). (D) Correlation of surface area, volume, and average thickness with detailed electrophysiological metrics. (E) Correlation of structural measures (BA1 through BA6) with electrophysiological metrics across specific frequency bands: alpha, low beta (Lbeta), high beta (Hbeta), and gamma. (F) Correlation of surface area, volume, and average thickness with electrophysiological metrics across specific frequency bands. BA = Broadman area, Surf = surface area, Vol = volume, Tick Avg= average thickness, LBeta = low beta band, Hbeta = high beta band, M1 = Motor cortex, PM = Premotor cortex, S1 = Primary somatosensory cortex, Sub-cort = subcortical region (Pallidum), Coherence-CC = cortico-cortical coherence, Coherence-M1 = cortico-subcortical coherence. Significant correlations are marked with asterisks in all panels.

We computed the correlation matrix for these subcategories across all 50 subjects using Pearson’s correlation coefficient. All combinations of cross-correlations were visualized using heatmaps, depicting individual correlations (Figure 3) and subgroup averages (Figure 4).

To assess the statistical significance of the observed correlations among the 36 variables, we performed permutation tests. The null hypothesis posited that no relationship exists between the variables, and any observed correlation is due to random chance. For the permutation tests, we independently permuted the data for each variable, effectively shuffling the values within each variable to disrupt existing associations while maintaining the original distribution. This process was repeated 1,000 times to generate a distribution of correlation coefficients under the null hypothesis.

The p-value for each observed correlation coefficient was calculated as the proportion of permuted correlation coefficients that were as extreme or more extreme than the observed value:

$$p_{\text{perm}}=\frac{\sum_{i=1}^{N}(\left|r_{\text{perm}}^{\left(i\right)}\right|\geq \left|r_{\text{obs}}\right|)}{N}$$

where

$p_{\text{perm}}$ represents the p-value obtained from the permutation tests, *i* denotes the permutation iteration, $r_{\text{perm}}^{\left(i\right)}$ is the correlation coefficient from the permuted data, and $r_{\text{obs}}$ is the observed correlation coefficient from the original dataset.

To adjust for multiple comparisons, we employed the False Discovery Rate (FDR) control using the Benjamini-Hochberg procedure. The permutation p-values were compared against thresholds determined by:

$${\text{Threshold}}_{\left(k\right)}=\frac{k}{m}\times \alpha$$

Where *k* is the rank of the p-value after sorting in ascending order,*m* is the total number of tests performed in the category, and α is the desired FDR, set to 0.05 in this study. We rejected all null hypotheses for which the p-values were less than or equal to their corresponding threshold.

#### Sparse Partial Least Squares Analysis

Recognizing that pairwise analysis does not account for associative effects among variables, we employed Sparse Partial Least Squares (SPLS) regression to explore multivariate associations between structural and electrophysiological measures (Wold, 2004; Abdi, 2010). SPLS is well-suited for capturing complex relationships between two data modalities, especially in high-dimensional datasets.

We utilized the SPLS implementation developed by (Mihalik et al., 2022) to train, regularize, and evaluate the model. SPLS optimizes the covariance between linear combinations of imaging and neurophysiological variables, producing weight vectors that indicate the contribution of each variable to the identified associations. The method includes sparsity constraints, enhancing model interpretability by selecting a subset of important features and focusing on the most relevant variables.

#### Model Training, Optimization, and Validation

To ensure generalizability and avoid overfitting, we employed a multiple holdout framework for optimizing regularization parameters and evaluating the SPLS models (Figure 2). The SPLS model was trained on an optimization set comprising 80% of the data, and the identified multivariate associations were assessed on a separate holdout set comprising the remaining 20%. The optimization set was further divided into training and validation subsets to fine-tune regularization parameters.

Optimal parameters were selected based on the ability to enhance generalization performance, determined by the out-of-sample correlation on the validation set, using a combined criterion of stability and generalizability. Generalizability was quantified as the average out-of-sample correlation on the validation and holdout sets. Stability was assessed by calculating the average similarity of the weight vectors (corrected overlap for SPLS) across different data splits.

To validate the robustness of the SPLS model, the entire process was repeated 10 times, following methodologies from previous studies (Monteiro et al., 2016; Baldassarre et al., 2017; Mihalik et al., 2019, 2022). This iterative approach allowed for the identification of structure-neurophysiology associations that are both stable and generalizable to new data.

To identify symptom-related factors using UPDRS scores, we performed stepwise multiple regression analyses using anatomical and physiological biomarkers weighted by SPLS-derived split weights. The stepwise approach was optimized using the Akaike Information Criterion (AIC) to select the best subset of predictors. Separate models were created for anatomical and physiological variables, and the analysis was conducted to determine which biomarkers significantly contributed to explaining UPDRS scores.

#### Essential Tremor (ET) Cohort Analysis

To evaluate whether the SPLS/CCA model and latent space were specific to PD pathophysiology, we tested the model on a cohort of 24 essential tremor (ET) patients. Data collection and the calculation of morphometric and neurophysiological variables were conducted in the same manner as for the main PD cohort. The SPLS weights from the best-performing split identified in the PD analysis were then applied to calculate the latent space for the ET patients. The correlation between anatomical and physiological latent scores was computed to determine whether the model captured meaningful associations in the ET cohort.

#### Editing and proofreading process

During the preparation of this work, the authors used AI-powered language tools for assistance with spelling and grammar. All AI-generated suggestions were manually reviewed and edited by the authors to ensure accuracy and clarity. The authors take full responsibility for the content of this publication.

## Results

The study included 50 PwPD, including 13 women with an average age of 64.36 years. Average Unified Parkinson’s Disease Rating Scale (UPDRS) III score was 37.73. Male participants had a mean age of 63.14 years and an average UPDRS score of 39.12, while female participants had a mean age of 67.85 years and an average UPDRS score of 36.39. Statistical analyses revealed no significant differences between male and female participants in terms of age (t-test; p = 0.062) or UPDRS III scores (t-test; p =0.721). For subjects list and demographics, refer to table 1.

### Pairwise Cross-Correlation Analysis

To explore the relationships between structural and neurophysiological biomarkers, we first calculated Pearson correlation coefficients for all possible pairs of these measures. Figure 3A illustrates these pairwise correlations across the two modalities. To assess the statistical significance of the observed patterns, we performed permutation tests by randomly shuffling the data for each subject and repeating the correlation analysis 1,000 times. Correlations that fell within the extreme 5% of the permutation distribution were considered significant and are plotted in Figure 3B.

Although individual pairwise comparisons appear scattered, grouping the variables into broader categories provides clearer insights. In Figure 3, the axes are organized based on morphometric measures (surface area, volume, and cortical thickness on the Y-axis) and neurophysiological measures (power, burst duration, burst amplitude, burst rate, and coherence on the X-axis). Notable patterns are apparent, such as a positive cluster for burst duration and a negative cluster for burst rate (Figure 3A). Permutation tests identified clusters of significant correlations, with red lines indicating boundaries where clusters exceeded three median absolute deviations from the center (Figure 3B).

Applying the Benjamini-Hochberg procedure to control the false discovery rate (α = 0.05), we highlighted significant correlations marked with asterisks in Figure 4. For example, Figure 4D shows a significant correlation between power and volume and surface area regardless of band. Another example is Figure 4C, it reveals a strong positive correlation between burst duration and cortical thickness, particularly in regions Brodmann areas (BA) 1 to BA6; however, these did not reach statistical significance. Similarly, burst rate exhibited significant correlations with cortical thickness and volume, especially across regions BA1 to BA3b (Figure 4C). All frequency bands demonstrated significant correlations with average cortical thickness (Figure 4F).

Despite revealing significant relationships between certain structural and neurophysiological measures, this pairwise analysis primarily provides a fragmented view of the associations. While clustering schemes were applied to identify patterns, they often showed weak convergence and failed to decisively uncover a fundamental relationship between the two modalities. This limitation underscores the need for more comprehensive methods to capture the interrelated nature of morphometric and neurophysiological variables.

### Associative Analysis Using Sparse Partial Least Squares

While pairwise analyses are effective for examining specific relationships between individual biomarkers and morphometric properties, they do not account for the overall associative effects of one modality on the other. To address this limitation, we employed Sparse Partial Least Squares (SPLS) regression to investigate the multivariate associations between structural and neurophysiological variables.

**Figure 5:**
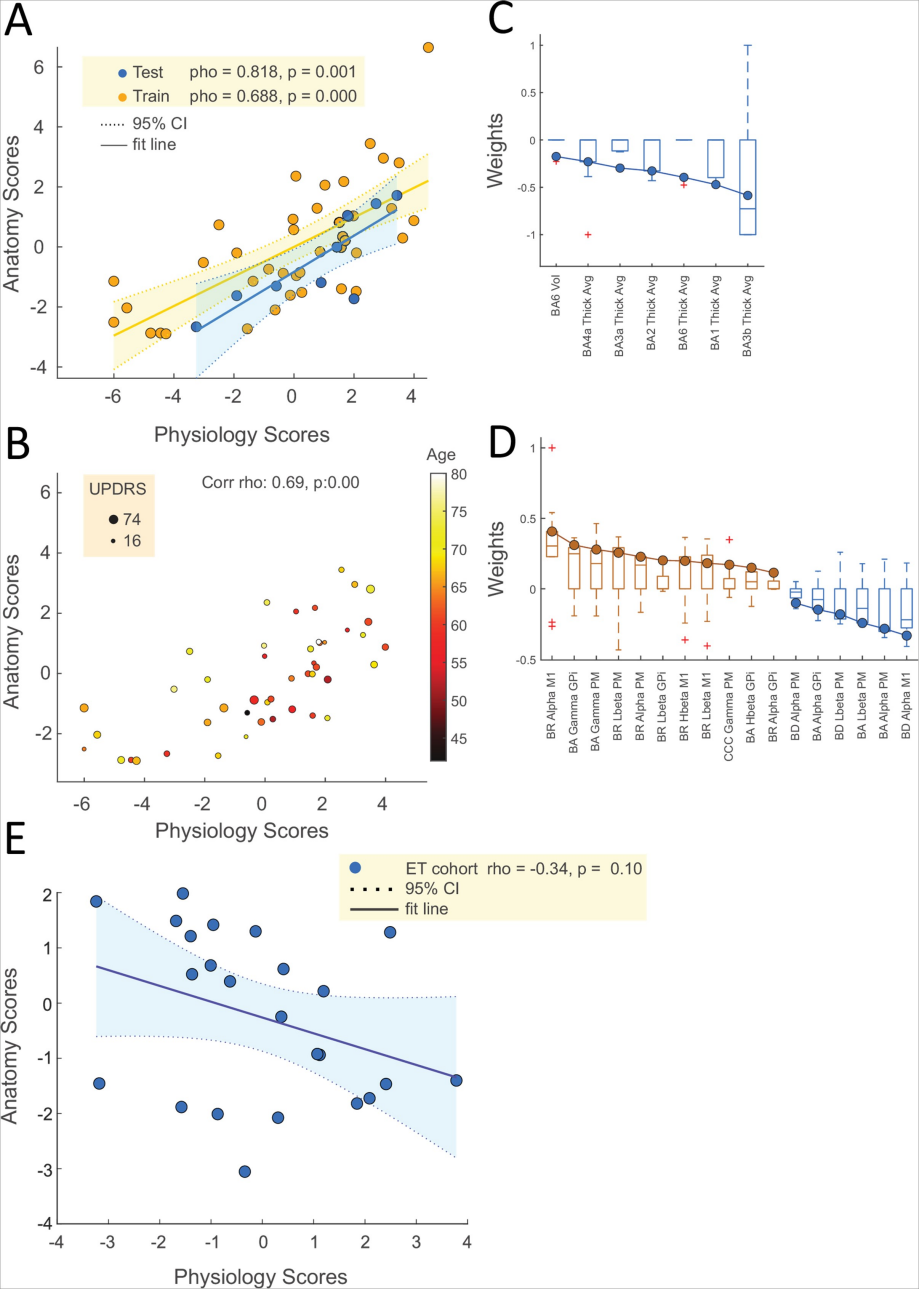
Canonical Correlation Between Structural and Neurophysiological Latent Scores and related weights. (A) Scatterplot illustrating the relationship between structural and neurophysiological latent scores in both the training (orange) and test (blue) sets. The fit line represents the linear regression, and the shaded area indicates the 95% confidence interval (CI). The strong correlation between these scores in both sets (train: ρ = 0.688, p < 0.001; test: ρ = 0.818, p = 0.001) highlights the robustness of the identified structure-neurophysiology association. (B) Scatterplot showing the relationship between structural and neurophysiological latent scores across all subjects, with color indicating patient age and size representing the Unified Parkinson’s Disease Rating Scale (UPDRS) scores. The correlation coefficient (ρ = 0.69, p < 0.001) demonstrates a significant association between the latent scores. (C) Boxplot of the weights for the structural variables contributing to the first latent dimension identified by SPLS. The structural variables include volume (Vol) and average thickness (Thick Avg) for Brodmann areas (BA) 6, 4a, 3a, 3b, 2, and 1. The negative weights indicate a strong negative association between cortical thickness and the neurophysiology latent dimension, particularly in the sensorimotor regions BA3b, BA1, BA6, BA2, and BA4a, suggesting that thinning in these regions is strongly linked to electrophysiological alterations in PD. (D) Boxplot of the weights for the neurophysiological variables contributing to the first latent dimension. The neurophysiological variables include burst rate (BR), burst duration (BD), and cortico-cortical coherence (CCC) across different frequency bands (alpha, low beta [Lbeta], high beta [Hbeta], gamma) and locations (M1, PM, GPi). Positive weights indicate a positive association with the structure’s latent dimension, with M1 alpha burst rate and gamma burst amplitude showing the strongest contributions. The negative weights, particularly in low beta burst duration and alpha burst duration, highlight their negative association with cortical thickness. (E) Scatterplot illustrating the relationship between structural and neurophysiological latent scores in ET control cohort. The fit line represents the linear regression, and the shaded area indicates the 95% confidence interval (CI). No significant correlation was found between structural and neurophysiological latent scores (ρ = −0.342, p = 0.102). BA = Broadman area, Vol = volume, Tick Avg= average thickness, LBeta = low beta band, Hbeta = high beta band.

Sparse Partial Least Squares (SPLS) is a multivariate statistical technique designed to identify and quantify relationships between two high-dimensional datasets. By maximizing the covariance between linear combinations of variables from each dataset, SPLS captures the underlying associations while imposing sparsity constraints. These constraints ensure that only the most relevant features contribute to the model, enhancing both interpretability and generalizability. In the context of this study, SPLS enables the discovery of latent dimensions that represent the interconnected dynamics between morphometric and neurophysiological variables, providing a holistic view of their associations. We analyzed the entire dataset using SPLS with a 20% holdout approach across 10 separate data splits. This analysis identified two significant latent dimensions representing associations between structure and neurophysiology. However, only the first latent dimension showed significant correlations between predicted and observed scores in the test sets (training set: ρ = 0.688, p < 0.001; test set: ρ = 0.818, p = 0.001; Figure 5A). Therefore, our focus is on the first latent dimension.

Table 2 summarizes the outcomes for the first latent dimension across all 10 splits. Five splits (bolded in Table 2) yielded significant results in the test samples that met the criteria for passing the omnibus hypothesis (Nichols and Holmes, 2001). To ensure robustness, we present results from the split that demonstrated the optimal balance of generalizability (assessed by out-of-sample correlation on the holdout set) and stability (assessed by the consistency of weights across optimization sets).

In this optimal split (test set: ρ = 0.688, p = 0.001; Table 2, row 9), the SPLS model revealed a strong negative correlation with average cortical thickness in regions BA3b, BA1, BA6, BA2, BA3a, and BA4a (Figure 5C). The most influential positive neurophysiological contributors were M1 alpha burst rate, globus pallidus internus (GPi) gamma burst amplitude, and PM gamma burst amplitude. Conversely, the strongest negative associations were observed with M1 alpha burst duration, PM alpha burst amplitude, and PM low beta burst amplitude (Figure 5D).

Figure 5A illustrates the correlation between the morphometric and neurophysiology latent variables in both the training and test sets for this optimal split. The high degree of alignment demonstrates the effectiveness of our cross-validation and regularization strategies. Consistent with SPLS methodology, the model produced sparse weight vectors, selecting between 3 and 51 features out of 92 possible neurophysiological variables (mean selection rate: 27.4% ± 1.4% SEM) and between 1 and 4 out of 36 possible morphometric variables (mean selection rate: 10.0% ± 1.9% SEM) across different splits. Notably, the best-performing models utilized a smaller number of features, underscoring the importance of feature selection for enhancing interpretability and generalizability.

The weight vectors for the significant neurophysiological measures (absolute weights > 0.1) are displayed in Figure 5D. These measures were the most influential contributors to constructing the latent variables, ultimately driving the observed correlations between the two modalities. For structural measures, the average cortical thickness in regions BA3b, BA1, BA6, BA2, BA3a, BA4a, and the volume of BA6 showed significant negative associations (Figure 5C). The optimal model included 29 non-zero weights out of 92 neurophysiological variables, primarily related to burst duration and amplitude within the alpha, gamma, and low beta bands. M1 alpha burst duration and M1 alpha burst rate were the most influential negative and positive weights, respectively (Figure 5D).

Although the second structure-neurophysiology latent space model reached statistical significance (best p = 0.036, ρ = 0.609), it did not reveal consistent sets of weights across the 10 splits and was not significant after Benjamini-Hochberg adjustment. Therefore, we do not present detailed findings for this model.

Scatterplots of the structure and neurophysiology latent scores, derived from applying the weights of the optimal model to all data points, visualize how the structure-neurophysiology relationship manifests across the latent space (Figure 5). The first latent variable showed a Pearson correlation coefficient of 0.649 (p < 0.001). In Figure 5B, the color and size of the data points represent the patients’ age and UPDRS scores, respectively. Subtle patterns suggest potential correlations between disease severity, age, and the latent space correlation effect.

### Assessment of Age and Disease Severity and Duration Effects

To investigate the potential influence of age and disease severity on the observed structure-neurophysiology relationship, we conducted partial correlation analyses. The results of partial correlation analysis between structure latent scores, neurophysiology latent scores, UPDRS scores, years since diagnosis and age are presented in Table 3. The correlation between neurophysiological and structural scores is strong and significant (ρ = 0.701, p < 0.001) while controlling for age, years since diagnosis (as a measure for disease duration), and UPDRS scores. This indicates that the observed canonical relationship between latent scores cannot be solely attributed to factors such as age or the chronic effects of disease progression.

It is noteworthy that we did not initially predefine PD-specific morphometric or electrophysiological biomarkers to include in the SPLS models. Therefore, while the strong correlation between latent scores is not entirely driven by chronic disease changes or age according to partial correlation analysis, these scores likely reflect PD-related changes as well as structure-neurophysiology correlations influenced by other factors. This hypothesis is supported by the lack of significant correlations between UPDRS scores and either structure or neurophysiology latent scores (ρ = −0.188, −0.139; p = 0.212, 0.356, respectively). We propose that the latent scores are influenced by a combination of variables, some directly related to disease severity and others independent of it.

To further disentangle these two sets of variables, we performed stepwise multiple regression analyses to identify factors linked to UPDRS scores. Using structure and neurophysiology biomarkers weighted by split weights, we applied a stepwise approach optimized with the Akaike Information Criterion (AIC) to select the best predictors. Both models converged on a small subset of variables, revealing significant correlations between UPDRS scores and specific biomarkers (r^2^ = 0.227, p = 0.004 for neurophysiology; r^2^ = 0.142, p = 0.037 for structure). The results of the stepwise regression analyses are highlighted in Tables 4 and 5, where variables identified as PD-related biomarkers are indicated.

Finally, to evaluate whether the model and latent space were specifically related to PD pathophysiology, we tested the approach on a cohort of 24 essential tremor (ET) patients. Morphometric and electrophysiological variables were calculated for the ET cohort, and the SPLS weights from the best-performing split reported in this study were applied to compute the latent space for these patients. The resulting correlation between structural and neurophysiological latent scores in the ET cohort did not show a significant association (Figure 5E, ρ = −0.342, p = 0.102). This control analysis suggests that the model and its identified latent dimensions are specifically tuned to the pathophysiological mechanisms underlying PD, as it failed to demonstrate similar correlations in a non-PD cohort.

## Discussion

### Multivariate and Pairwise Analyses Reveal Structural-Functional Associations

Utilizing both traditional pairwise analyses and advanced multivariate methods, we uncovered an underlying relationship that extends beyond isolated pairwise correlations between cortical morphometry and neurophysiological variables within the motor cortico-basal ganglia network that is specific to PwPD.

Pairwise cross-correlation analysis revealed significant relationships between specific electrophysiological measures and structural features. Notably, prolonged burst durations and cortical-subcortical coherence were positively correlated with cortical thickness and surface area, suggesting that differences in cortical morphometry may contribute to changes in oscillatory dynamics. Conversely, increased burst rates and cortico-cortical coherence, specifically between the primary motor and either premotor or primary somatosensory cortex, were negatively correlated with cortical thickness and volume. This negative correlation could reflect two seemingly paradoxical scenarios: increased coherence despite physical shrinkage, or decreased coherence with relative physical expansion. In both cases, a compensatory mechanism becomes plausible. When cortical volume or thickness decreases, the remaining pathways may enhance coherence or signal dynamics to maintain information flow. Conversely, in scenarios with relative increases in cortico-cortical pathways, a reduction in coherence may serve to regulate synchronized activity and prevent excessive oscillation.

### Compensatory Mechanisms and Latent Multivariate Associations

While there remains a logical leap in directly attributing this pattern to compensation, it is reasonable to hypothesize that coherence and signal dynamics (represented here by bursting) adjustments may serve as a compensatory mechanism, as these can be more readily modulated by altering the firing rates of neurons. These relationships were particularly evident in sensorimotor regions corresponding to Brodmann areas BA1 to BA6. The strongest associative effects were associated with low beta, alpha and gamma oscillatory activity in PM, M1, and subcortical regions, the globus pallidus internus (GPi). This supports the hypothesis that abnormal alpha and beta activity reflects underlying structural degeneration. To validate this hypothesis, we applied the model to an essential tremor (ET) cohort, which failed to demonstrate the same level of significant correlations, further reinforcing the specificity of the observed associations in PD.

### Regional Contributions and Disease Progression

The analysis also identified BA1 and BA3b thickness as key contributors to the observed associations, suggesting that early changes in these regions may play a role in perpetuating the cycle of electrophysiological abnormalities and motor symptoms in PD. Notably, our partial correlation analysis revealed that disease duration was correlated with latent electrophysiology scores but not with structure scores. While this might seem counterintuitive, it implies that structural changes do not progress gradually throughout all stages of the disease. This observation aligns with previous structural studies showing that PD-related cortical thinning occurs more rapidly during the early stages even before clinical manifestations (Wilson et al., 2019; Park et al., 2022). Our findings remained significant after controlling for potential confounding factors such as age, disease duration, and disease severity as measured by UPDRS scores. This indicates that the observed correlations are more likely to be reflective of disease-specific pathophysiological processes rather than being artifacts of generalized aging, the chronic effects of prolonged illness, or the accumulated impact of years of motor impairment. Furthermore, the lack of correlation between the magnitude of the structure-neurophysiology link and variables like age, disease duration, and UPDRS scores suggests that the factors driving this relationship are distinct from conventional biomarkers typically associated with disease severity and progression. This divergence highlights the potential for uncovering novel biomarkers and previously unexplored aspects of PD pathophysiology. However, further fundamental research is required to fully elucidate the mechanistic relationship. The consistency of these findings across multiple data splits and cross-validation procedures further underscores their validity and potential clinical relevance.

### Structural-Functional Integration and Biomarker Specificity

Our results strongly support the hypothesis that dynamic, complex electrophysiological factors are closely correlated with structural brain alterations in PD. Specifically, burst duration in motor, premotor, and pallidal regions, as well as burst rates (Table 4), combined with cortical volume and average thickness (Table 5) in the structural modality, indicate a fundamental relationship between brain structure and electrophysiology. While other studies have captured similar underlying mechanisms using diverse methods and modalities, differences in methodologies and measures make direct, one-to-one comparisons challenging, underscoring the existence of such fundamental principles rather than specific metrics (Zarkali et al., 2021; Chen et al., 2023).

Our further analysis using stepwise linear regression highlights that only a subset of these biomarkers—namely alpha and beta range burst rates in M1, PM, and pallidal regions, along with cortical thinning in the somatosensory and premotor cortices—were significantly linked to PD severity. This finding suggests that while both disease-related and other biomarkers contribute to the overall latent variables, the identified subset may be particularly relevant for disease progression and symptom manifestation.

### Oscillatory Dynamics, Regional Specificity, and Clinical Implications

Importantly, the convergence of structural and functional biomarkers into shared highly correlated latent variables highlights a key observation: stationary structural changes serve as a foundational substrate for the dynamic alterations observed in electrophysiological activity. In the context of PD, atrophy of specific cortical regions may disrupt normal oscillatory patterns, particularly in the alpha and beta bands, leading to the emergence of abnormal and compensatory oscillatory activity. We propose that compensatory mechanisms are particularly likely during the early stages of PD (Pollok et al., 2013; Wilson et al., 2019; Park et al., 2022), since structural alterations are present but motor signs and symptoms have yet to manifest. The involvement of alpha and low beta oscillations in PM, M1, and subcortical regions further underscores the complexity of electrophysiological changes in PD. Alpha oscillations are crucial for sensorimotor processing and cortical excitability (Pfurtscheller et al., 1996; Hirschmann et al., 2019), while beta oscillations are strongly associated with motor impairments in PD (Brown, 2007; Van Wijk et al., 2017; Malekmohammadi et al., 2018a, 2018b). Moreover, our results align with previous neuroimaging studies reporting cortical thinning in sensorimotor areas of PD patients (Zhang et al., 2018; Perinelli et al., 2021). The observed associations between cortical thinning and electrophysiological alterations provide a potential explanation for the structural-functional disconnects (Zarkali et al., 2021) often observed in PD. Our results are consistent with studies suggesting that combined structural and network-level metrics offer critical insights into PD-related network reorganization (Bergamino et al., 2023).

By focusing on cortical thickness and a comprehensive range of electrophysiological biomarkers, our study extends previous findings and highlights the role of sensorimotor regions as key areas of structural-functional integration in PD. Understanding the link between electrophysiological biomarkers and cortical morphometric changes has significant clinical implications. These robust associations could serve as a foundation for developing comprehensive diagnostic tools and improving disease staging and progression monitoring. Structural measures that strongly correlate with electrophysiology, such as cortical thinning in specific regions, may also serve as biomarkers for the early detection and assessment of PD severity.

These findings suggest that PD progression is driven not only by dopaminergic deficits but also by concurrent structural and electrophysiological changes (Wiesman et al., 2024). A multimodal approach that integrates neuroimaging and electrophysiology holds great promise for improving the diagnosis, monitoring, and treatment of PD. Such an approach could facilitate personalized therapeutic strategies aimed at mitigating both structural degeneration and abnormal oscillatory activity, ultimately improving patient outcomes and quality of life.

### Limitations and Future Directions

The cross-sectional design of this study precludes causal inferences about the relationship between electrophysiological alterations and morphometric differences. Longitudinal studies are needed to determine whether these markers can predict disease progression and response to treatment.

The challenges associated with collecting electrophysiological data during deep brain stimulation (DBS) surgery limited the number of cases and regions included in the study, which consequently restricted the range of variables analyzed. We chose to focus on cortical structural measures, particularly within the sensory-motor cortex, as these regions have been relatively less studied in terms of their structural correlations with electrophysiology and were a central focus of our research. In contrast, subcortical structures such as the pallidum and STN require more precise structural methods for segmentation and volumetric analysis to ensure accurate correlation with electrophysiological data. Investigating these subcortical gray nuclei will require a dedicated study utilizing advanced volumetric and shape analysis techniquesAdditionally, the sample size, although adequate for SPLS analysis, limits the generalizability of the findings. Larger, multicenter studies could enhance the robustness of the results and allow for subgroup analyses, such as exploring differences based on disease subtypes, treatment regimens, or genetic factors.

A limitation of our control analysis is that the ET cohort lacked basal ganglia recordings, so comparisons to PD relied solely on cortical data. This mismatch reduces our ability to assess cortico–basal ganglia couplings in ET. Nevertheless, the absence of a similar cortical-only latent relationship in ET indicates our findings are not generic cortical effects but align with PD-specific pathophysiology.

The significant associations between specific electrophysiological biomarkers and cortical thinning in sensorimotor regions underscore the interconnected nature of structural and functional alterations in PD.

Overall, the integration of structural and functional data presents a promising avenue for advancing our understanding of PD and gaining greater insights into pathoetiologic mechanisms of disease.

## Supplementary Files

This is a list of supplementary files associated with this preprint. Click to download.

- table1.docx
- table2.docx
- table3.docx
- table4.docx
- tabl5.docx

## Figures and Tables

**Table 1:** Patient demographics

**Table 2.** Summary of Sparse Partial Least Squares (SPLS) Model Performance Across Different Data Splits. The table presents the results of the SPLS analysis across ten different data splits. For each split, the training and test set correlations are shown alongside the corresponding test p-values, the number of selected physiology features, and the number of selected anatomy features. The final column lists the Benjamini-Hochberg (BH) adjusted p-values for significance. Significant correlations between anatomy and physiology latent dimensions are highlighted in bold. The results indicate the robustness and generalizability of the model, with certain splits (e.g., 2, 6, 7, and 9) demonstrating particularly strong and significant associations.

**Table 3.** Partial Correlation Coefficients Between Physiological, Anatomical, UPDRS Scores, and Age Variables. This table presents the partial correlation coefficients among the physiological scores, anatomical scores, UPDRS scores, Years since diagnosis and age, controlling for the influence of other variables. Significant correlations at the BH corrected alpha of 0.05 are highlighted in bold.

**Table 4.** SPLS electrophysiology weights. Nonzero Weights of SPLS electrophysiology. The bold rows are factors that have shown a significant correlation with UPDRS III scores using stepwise linear regression. Abbreviations: Sub-Cort is subcortical, Lbeta is low beta, Lbeta is high beta, CC is cortico-cortical

**Table 5.** SPLS anatomy weights. All nonzero Weights of SPLS Anatomy. The bold rows are factors that have shown a significant correlation with UPDRS III scores using stepwise linear regression. Abbreviations: Thick is thickness, Avg is average.

## Data Availability

The datasets analyzed during the current study are available in the Brain Integrated Resource for Human Anatomy and Intracranial Neurophysiology(B(RAIN2) repository(Alijanpourotaghsara et al., 2025), https://doi.org/10.18120/zmah-2816.

## Code availability

The underlying code for this study is not publicly available but may be made available to qualified researchers on reasonable request from the corresponding author.
